# Spleen tyrosine kinase mediates the γδTCR signaling required for γδT cell commitment and γδT17 differentiation

**DOI:** 10.3389/fimmu.2022.1045881

**Published:** 2023-01-12

**Authors:** Ryunosuke Muro, Tomoya Narita, Takeshi Nitta, Hiroshi Takayanagi

**Affiliations:** ^1^ Department of Immunology, Graduate School of Medicine and Faculty of Medicine, The University of Tokyo, Tokyo, Japan; ^2^ Department of Pharmacotherapeutics, Research Institute of Pharmaceutical Sciences and Faculty of Pharmacy, Musashino University, Tokyo, Japan

**Keywords:** γδT cell, TCR signal, SYK (spleen tyrosine kinase), IL-17, thymus

## Abstract

The γδT cells that produce IL-17 (γδT17 cells) play a key role in various pathophysiologic processes in host defense and homeostasis. The development of γδT cells in the thymus requires γδT cell receptor (γδTCR) signaling mediated by the spleen tyrosine kinase (Syk) family proteins, Syk and Zap70. Here, we show a critical role of Syk in the early phase of γδT cell development using mice deficient for Syk specifically in lymphoid lineage cells (Syk-conditional knockout (cKO) mice). The development of γδT cells in the Syk-cKO mice was arrested at the precursor stage where the expression of Rag genes and αβT-lineage-associated genes were retained, indicating that Syk is required for γδT-cell lineage commitment. Loss of Syk in γδT cells weakened TCR signal-induced phosphorylation of Erk and Akt, which is mandatory for the thymic development of γδT17 cells. Syk-cKO mice exhibited a loss of γδT17 cells in the thymus as well as throughout the body, and thereby are protected from γδT17-dependent psoriasis-like skin inflammation. Collectively, our results indicate that Syk is a key player in the lineage commitment of γδT cells and the priming of γδT17 cell differentiation.

## Introduction

γδT cells exert multiple functions while acting as innate effector cells that produce cytokines and/or kill infected or malignant cells. Recently, accumulating evidence has suggested the versatile roles played by a subset of IL-17-producing γδT cells (γδT17 cells) in inflammatory disorders (1), tumor progression (2), tissue regeneration (3–5), and fibrosis (6) as well as affording protection from bacterial and fungal infection (7).

The development of both αβT and γδT cells occurs in the thymus. αβT lineage cells undergo a differentiation process through CD4^−^ CD8^−^ (double negative, DN), CD4^+^ CD8^+^ (double positive, DP), and CD4^+^ CD8^-^ (CD4 single positive, CD4SP) or CD4^-^ CD8^+^ (CD8SP) thymocytes, during which the T cell receptor (TCR) β and α chains are sequentially rearranged and immunocompetent yet self-tolerant αβTCRs are selected upon the interaction with self-peptide/major histocompatibility complex (8). In contrast, γδT lineage cells originate from DN thymocytes, in which the TCRγ and δ chains are simultaneously rearranged (9). Unlike the well-studied mechanisms of αβT cell development, the claim that the γδTCR-ligand interaction is required for γδT cell differentiation is still controversial. The effector function of γδT cells is reportedly determined in the thymus, where ligand-dependent strong or ligand-independent weak γδTCR signals lead to IFNγ-producing (γδT1) or IL-17-producing γδT (γδT17) cells, respectively (10–12). The effector function of γδT cells has a profound connection with TCR-Vγ repertoire. The γδT cells expressing Vγ4 or Vγ6 cells preferentially produce IL-17, while Vγ1, Vγ5, or Vγ7 γδT cells mainly produce IFNγ. It is also known that these distinct γδT cell subsets home to lymphoid and mucosal tissues depending on the TCR-Vγ chains they expressed (e.g., Vγ6 cells in the mucosal epithelia such as dermis and lung, Vγ7 cells in the intestine, Vγ5 cells in the epidermis, and Vγ1/Vγ4 cells in the lymphoid tissues).

Immune receptors including the TCR require the Spleen tyrosine kinase (Syk) family protein tyrosine kinases, Syk or Zap70, to activate intracellular signaling events (13). Zap70 is uniquely required for αβTCR signaling and αβT cell development (14). In contrast, Syk is involved in signal transduction in other receptor types such as the B cell receptor, Fc receptors, C-type lectin receptors, and erythropoietin receptors, and play critical roles in various physiological and pathological conditions (13, 15). In the early stage of αβT cell development, Zap70 and Syk play a redundant role in pre-TCR signaling (16, 17).

We previously have shown that Syk as well as Zap70 are activated upon γδTCR signaling and that mice lacking Syk but not Zap70 exhibit reduced γδTCR signaling in γδT cells (18). These results with genetically or pharmacologically manipulated T-progenitor cells demonstrated that Syk-mediated γδTCR signal activates both the Lat/MAPK and PI3K/Akt pathways so as to induce γδT17 cell differentiation. However, these findings were obtained using γδT cells from fetal or neonatal mouse thymus, since systemic Syk-deficient mice are perinatally lethal due to an abnormality in lymphatic vascular development (19, 20). Thus, the contribution of Syk to γδT cell development in the adult stage is not yet fully understood.

In this study, we generated lymphoid lineage-specific Syk-deficient mice (hereafter called Syk conditional knockout mice: Syk-cKO mice). The results clearly demonstrate that, in adult mice, Syk is required for the thymic development of γδT cells, but not αβT cells. A substantial fraction of thymic γδT cells in Syk-cKO mice are in an immature state since they express Rag recombinases as well as the pTα chain, indicating that Syk drives γδT-cell lineage commitment. Furthermore, Syk-cKO mice completely lacked the development of γδT17 cells in the thymus and were largely protected from imiquimod-induced psoriasis-like dermatitis. These results offer conclusive evidence that Syk-mediated TCR signaling is critical for both γδT-cell lineage commitment and γδT17 cell priming.

## Materials and methods

### Animals

C57BL/6N mice were purchased from SLC Japan (Shizuoka, Japan). Syk^flox^ mice (21) were purchased from the Jackson laboratory (No. 017309). Cd127-Cre mice were described previously (22). The mice were maintained under specific pathogen-free conditions in our animal facility and euthanized by overdose of inhalational anesthetics. All animal experiments were performed with the approval of the Institutional Animal Care and Use Committee of the University of Tokyo, and conducted in accordance with institutional procedures.

### Cell preparation

Thymocytes, splenocytes, and lymph node cells were prepared by homogenizing the organs. Lung and skin cells were prepared by digesting minced tissues with 0.2% collagenase D (Roche) and 0.01% DNase I (Roche) at 37°C for 30 minutes. The digested tissues were disrupted by using a syringe and 18-gauge needle. The enzymatic reaction was stopped by adding PBS containing 2 mM EDTA and 2% FCS. The homogenized or digested cells were passed through 100-μm nylon mesh to obtain single cell suspensions. To prepare IEL from the small intestine, gut fragments were cut open and incubated with PBS containing 30 mM EDTA at 4°C for 30 minutes. Then the incubated fragments were washed and shaken in cold PBS to collect the small intestine epithelial fraction. The lymphocyte fractions were isolated from the small intestine using a 40-80% Percoll gradient (GE Healthcare).

### Flow cytometry analysis

FACSCantoII (BD Bioscience) was used for the flow cytometry analysis. Cells were treated with an Fc blocker (anti-CD16/32, clone 2.4G2, TONBO Bioscience) prior to cell surface staining. The cells were stained with the indicated antibodies at the final concentration of 1 to 2 μg/ml. To exclude dead cells, 7-aminoactionomycin D (7AAD) was added to the cell suspension at the final concentration of 0.3 μg/ml. For the intracellular staining, a Foxp3 Staining Buffer Set (eBioscience) was used according to their manufacturer’s protocol. The antibodies used for flow cytometry were as follows: CD4 (GK1.5), CD5 (53-7.3), CD8α (53-6.7), TCRβ (H57-597), CD3 (17A2), B220 (RA3-6B2), Ly6C (HK1.4), NK1.1(PK136), CD45RB (C363-16A), CD44 (IM7), CD25 (PC61), CD24 (30-F1), CD27 (LG.3A10), Gr-1 (RB6-8C5), TCRδ (GL3), TCR-Vγ1 (2.11), TCR-Vγ4 (UC3-10A6), TCR-Vγ5 (536), TCR-Vγ7 (GL1), IL-17A (TC11-18H10.1), IFN-γ (XMG1.2), phospho-ERK (p-ERK, 197G2) and phospho-AKT (p-Akt; D9E). The monoclonal antibody 17D1, specific for TCR-Vγ6/Vδ1 and TCR-Vγ5/Vδ1, was provided by Robert E. Tigelaar (Yale University, New Haven, Connecticut, USA) and used as described previously (23). Anti-Vγ7 monoclonal antibody (GL1) was provided by the late professor Leo Lefrançois (University of Connecticut Health Center, Farmington, Connecticut, USA) (24).

### Cell stimulation

For the induction of the TCR signal, 1.0 ×10^7^ thymocytes were resuspended in RPMI 1640 complete medium (25) that had been prewarmed at 37°C for 5 minutes, then an equal volume of prewarmed complete medium containing biotinylated anti-CD3ε antibody (60 μg/ml; 145-2C11; Biolegend) and streptavidin (21 μg/ml; SouthernBiotech) was added. At the indicated timepoint, 4% paraformaldehyde was added to stop the stimulation. For cytokine production, cells were incubated in RPMI 1640 complete medium in the presence of phorbol myristate acetate (PMA; 2.5 ng/ml), ionomycin (1 μg/ml) and brefeldin A (1 μg/ml) at 37°C for 4 hours.

### Quantitative mRNA analysis

Total RNA was extracted from FACS-sorted cells using the RNeasy Micro Kit (QIAGEN) and reverse transcribed with Superscript III (Invitrogen, Thermo Fisher Scientific). Quantitative PCR was performed with SYBR Premix ExTaq (TaKaRa) and the StepOne Real-Time PCR System (Life Technologies, Thermo Fisher Scientific). The results were normalized to the Gapdh expression levels. The primer sequence used in this study is described in Supplementary Material.

### Imiquimod-induced dermatitis

Daily application of 5 mg Beselna cream (5% IMQ; Mochida Pharmaceutical) or an equal amount of control vaseline cream (Wako) on mouse ears was performed to induce psoriasis-like dermatitis. Ear thickness was measured with a micrometer. On day 5, cervical lymph nodes were isolated and analyzed by flow cytometry.

### Statistical analysis

All data are represented as the mean ± SEM. Differences with a P-value of <0.05 were considered significant. For statistical analysis, Prism 7 software (GraphPad Software Inc.) was used. Two-tailed unpaired t-tests were used for comparing two groups, and one-way ANOVA tests were used when comparing three groups or more.

## Results

### Impaired thymic γδT cell development in Syk-cKO mice

We crossed Syk^flox^ mice with Cd127-Cre mice that express Cre recombinase under the control of the endogenous CD127 (IL-7R) promoter to specifically delete the Syk gene in lymphoid cells (21, 22). The generated Cd127-Cre Syk^flox/flox^ (Syk-cKO) mice are assumed to lack the expression of Syk in both αβT cells and γδT cells as well as other lymphoid lineage cells, including B cells, natural killer (NK) cells, and innate lymphoid cells.

**Figure 1:**
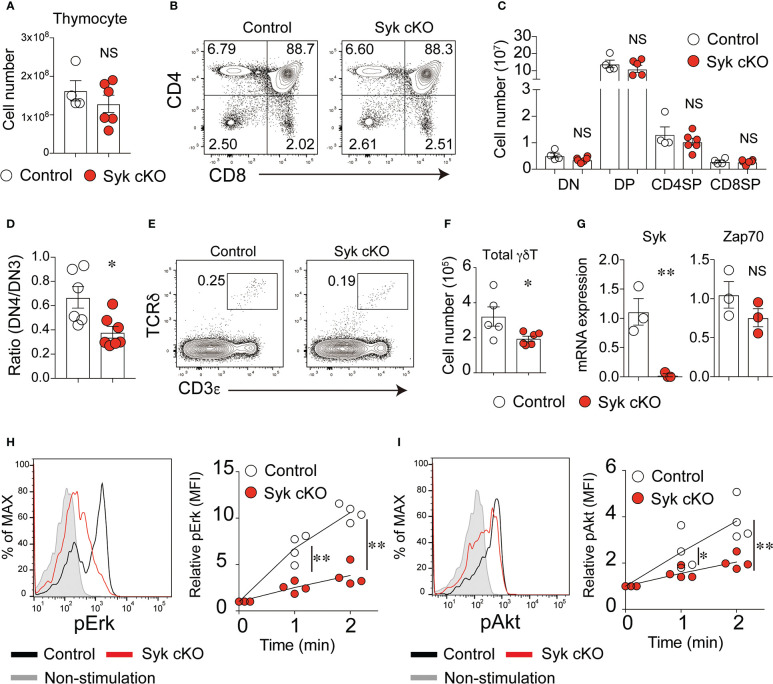
Syk is required for the development of γδT cells but not of αβT cells. **(A)** The number of thymocytes from 5-week-old control mice (Cd127-cre Syk flox/wt) or Syk-cKO mice (Cd127-cre Syk flox/flox). Each circle indicates an individual mouse. **(B)** Flow cytometric profiles for CD4 and CD8α expression in the total thymocytes from the indicated mice. **(C)** The graph indicates the number of DN, DP, CD4SP and CD8SP cells. **(D)** The ratio of CD25^lo^ CD44^lo^ DN (DN4) to CD25^hi^ CD44^lo^ DN (DN3) cells is shown. **(E)** Flow cytometric profiles for CD3ε and TCRδ expression in the total thymocytes from the indicated mice. **(F)** The number of thymic γδT cells is shown. **(G)** mRNA expression of Syk and Zap70 in sorted thymic γδT cells from control and Syk-cKO mice. Gene expression was normalized to *Gapdh* mRNA. **(H, I)** The phosphorylation of Erk **(H)** and Akt **(I)** induced by anti-CD3ε stimulation in thymic γδT cell. Representative flow cytometric profiles of the phospho-Erk (pErk) and phospho-Akt (pAkt) levels after 2-minutes of stimulation are shown. Graphs indicate the changes in the mean fluorescence intensity (MFI) of pErk and pAkt in the stimulated cells relative to non-stimulated cells. All data represent the mean ± SEM of at least two independent experiments. *P < 0.05 and **P < 0.01, by unpaired t-test. NS, not significant.

At the age of 4 to 5 weeks, Syk-cKO mice had a normal number of thymocytes without affecting the frequency of DN, DP, CD4SP or CD8SP cells (**Figures 1A**–**C**). In the spleen and lung, the number of B cells was markedly reduced in Syk-KO mice, while the number the NK cells was not significantly altered (**Figure S1A** in Supplementary Material). Consistent with previous findings that Syk controls pre-TCR signaling (17), Syk-cKO mice exhibited increases in the frequency of DN3 cells and decreases in the frequency of DN4 cells, resulting in a partial reduction of the DN4/DN3 ratio (**Figure 1D** and **Figure S2**). Both the frequency and number of DN1 (CD25^–^ CD44^+^) cells were significantly reduced in Syk-cKO mice. This can be explained by the impaired development of B cells in Syk-cKO mice, as the CD25^–^ CD44^+^ cell population contains many thymic B cells (26, 27). The number of DP, CD4SP, and CD8SP cells was comparable between the Syk-cKO mice and control mice, indicating that Syk-deficiency has no apparent effect on αβT cell development.

In contrast, the number of CD3^+^ TCRδ^+^ γδT cells was significantly reduced in the thymus of Syk-cKO mice (**Figures 1E, F**). The cell surface expression of TCRδ and CD3ε in Syk-deficient γδT cells was lower than that in control cells (**Figure S3**). In the thymic γδT cells isolated from Syk-cKO mice, the expression of functional Syk mRNA was totally absent, whereas the expression of Zap70 was comparable to that in control mice (**Figure 1G**). We measured the phosphorylation of Erk and Akt proteins in γδT cells upon anti-CD3ε stimulation to examine the effect of Syk deficiency on γδTCR signaling. Despite a normal expression of Zap70, TCR signal-induced phosphorylation of Erk and Akt was significantly diminished in Syk-cKO γδT cells (**Figures 1H, I**). These results indicate that Syk is required for optimum γδT cell development and γδTCR signaling.

### Failure of γδ-selection in Syk-cKO mice

**Figure 2:**
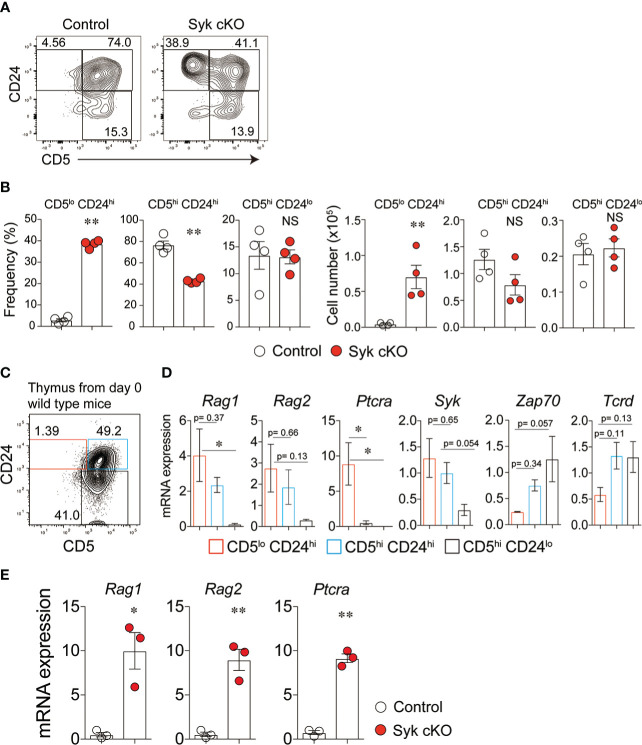
Impaired γδ-selection in Syk-cKO mice. **(A, B)** Flow cytometric profiles of CD5 and CD24 expression in total γδT cells from thymuses of control or Syk-cKO mice **(A)** and the number and frequency of the indicated γδT cell subset are shown **(B)**. **(C)** Flow cytometric profiles of CD5 and CD24 expression in thymic γδT cells from 0-day-old wild-type mice. **(D)** mRNA expression of the *Rag1*, *Rag2*, *Ptcra* (pTα), *Syk*, *Zap70* and *Tcrd* in γδT cell subsets shown in **(C)**. Gene expression was normalized to *Gapdh*, and those in total thymic γδT cells were arbitrarily set to 1. **(E)** mRNA expression of *Rag1*, *Rag2* and *Ptcra* in thymic γδT cells from the indicated mice (n = 3). Gene expression was normalized to *Gapdh*. All data represent the mean ± SEM. In **(A)** and **(B)**, data are obtained from two independent experiments. *P < 0.05 and **P < 0.01, by 1-way ANOVA **(D)** or unpaired t-test **(B, E)**. NS, not significant

We hypothesized that the dampened γδTCR signaling in Syk-cKO mice influences the differentiation processes of γδT cells. To evaluate the effect of Syk deficiency on the thymic γδT cell differentiation, we examined cell surface expression of CD5 and CD24; the former is a widely used indicator of TCR signaling (28), whereas the latter is expressed by immature T cells (29). CD5^lo^ CD24^hi^ γδT cells in control mice were only 2.9 ± 0.74% of total γδT cells, whereas this population was drastically increased to 38.5 ± 0.9% in Syk-cKO mice (**Figures 2A, B**). The frequency of CD5^hi^ CD24^hi^ γδT cells was 76.7 ± 3.8% and 42.0 ± 1.0% of the total γδT cells in the control and Syk-cKO mice, respectively. The frequency of CD5^hi^ CD24^lo^ γδT cells was not significantly different between the control and Syk-cKO mice.

To characterize the γδT cell subpopulations defined by CD24 and CD5 expression, we examined the gene expression profiles in γδT cell populations isolated from neonatal wild-type mouse thymus (**Figure 2C**). CD5^lo^ CD24^hi^ γδT cells exhibited high-level expression of recombination activating gene 1 (*Rag1*), *Rag2* and *Ptcra* (pTα) (**Figure 2D**), indicating that CD5^lo^ CD24^hi^ γδT cells retain the potential to rearrange TCR genes and to differentiate into the αβT-cell lineage. The expression of these genes was down-regulated in CD5^hi^ CD24^hi^ and CD5^hi^ CD24^lo^ γδT cells. Thus, the CD5^lo^ CD24^hi^ cells represent γδT precursors that complete γδTCR rearrangement yet are uncommitted to the γδT cell lineage. Indeed, γδT cells isolated from Syk-cKO mice displayed a markedly higher expression of *Rag1*, *Rag2* and *Ptcra* compared with those from control mice (**Figure 2E**). In general, the expression of Syk and Zap70 is inversely regulated during the development of thymic T cells: Syk is highly expressed until DN3 thymocyte stages and downregulated after β-selection, while Zap70 expression is induced after β-selection and maintained thereafter (9). Our results showed that the expression of Syk is highest in CD5^lo^ CD24^hi^ γδT cells but gradually decreased in CD5^hi^ CD24^hi^ and CD5^hi^ CD24^lo^ γδT cells (**Figure 2D**). On the other hand, the expressional pattern of Zap70 was opposite to that of Syk. These results indicate that Syk is required for the earliest step in γδT cell differentiation (previously referred to as “γδ-selection”) the step at which γδT precursors are committed towards the γδT cell lineage at the expense of a more limited αβT cell lineage potential (9).

### Syk is essential for the development of γδT17 cells

**Figure 3:**
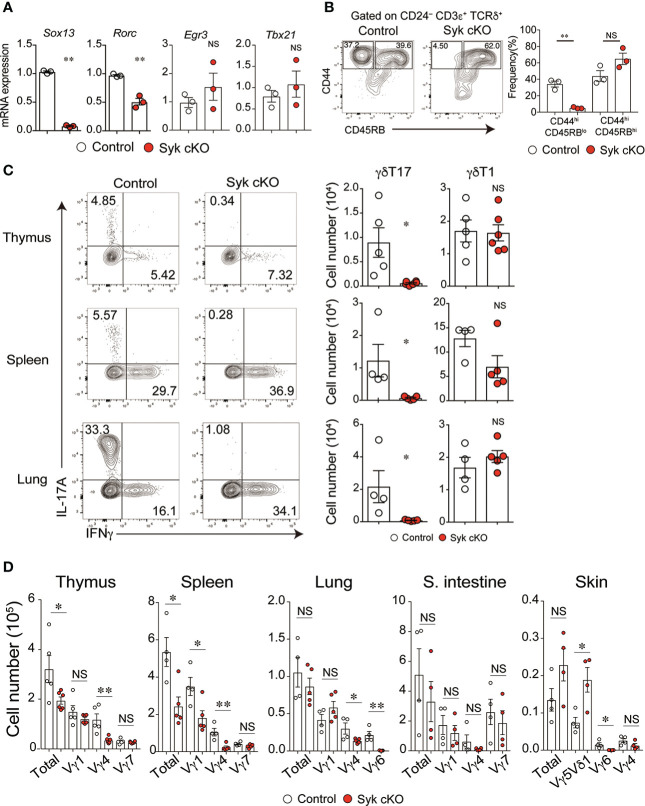
The impaired development of γδT17 cells but not γδT1 cells in Syk-cKO mice. **(A)** mRNA expression of *Sox13*, *Rorc*, *Egr3* and *Tbx21* in thymic γδT cells from control (Cd127-cre Syk flox/wt, n = 3) or Syk-cKO mice (Cd127-cre Syk flox/flox, n = 3). Gene expression was normalized to *Gapdh*. **(B)** Flow cytometric profiles of CD44 and CD45RB expression in CD24^lo^ γδT cells from thymuses of control or Syk-cKO mice. The graph indicates the frequency of the indicated γδT cell subset. **(C)** Intracellular staining for IL-17A and IFNγ production in thymic, splenic and lung γδT cells from the indicated mice (n = 5 to 6). Cell staining was performed after a stimulation with PMA and ionomycin. The number of γδT17 and γδT1 cells is shown. **(D)** Graphs show the number of Vγ1^+^, Vγ4^+^, Vγ5Vδ1^+^, Vγ6^+^ and Vγ7^+^ γδT cells as well as total γδT cells in the indicated tissues (n = 4 to 6). All data represent the mean ± SEM. In **(B–D)**, three independent experiments were performed. *P < 0.05 and **P < 0.01, by unpaired t-test. NS, not significant

We next examined the effector function of γδT cells in Syk-cKO mice. The mRNA expression of *Rorc* and *Sox13*, essential transcriptional factors for the γδT17 cell differentiation program, was significantly reduced in Syk-deficient γδT cells, whereas *Egr3* and *Tbx21*, both of which promote thymic γδT1 cell differentiation, were normally expressed (**Figure 3A**) (30). To investigate the functional difference of Syk-deficient thymic γδT cells, we analyzed cell surface expression of CD44 and CD45RB that segregates mature γδT cells into γδT17 cells and γδT1 cells (10). The frequency of CD45RB^lo^ CD44^hi^ CD24^lo^ γδT cells that correspond to γδT17 cells was 34.3 ± 3.6% and 5.2 ± 0.6% in control and Syk cKO mice, respectively (**Figure 3B**). In contrast, the frequency of CD45RB^lo^ CD44^hi^ CD24^lo^ cells (mainly γδT1 cells) was statistically unchanged (**Figure 3B**). Consistent with this, production of IL-17 upon PMA/ionomycin stimulation was barely detected in thymic γδT cells from Syk-cKO mice (**Figure 3C**). These mice also exhibited a complete loss of γδT17 cells in the spleen and lungs, indicating a pivotal role for Syk in the development of γδT 17 cells. In contrast, the number of γδT1 cells was normal in the thymus and lungs of Syk-cKO mice, although it was partially (not significantly) reduced in the spleen (**Figure 3C**). Given that the effector function of γδT cells is correlated with TCR-Vγ chains (31), we examined the TCR-Vγ repertoire in various tissues. The number of Vγ4^+^ and Vγ6^+^ γδT cells (mostly γδT17 cells) was substantially reduced in the thymus, spleen, lung, small intestine and skin of Syk-cKO mice, while the number of Vγ1^+^, Vγ5^+^ and Vγ7^+^ γδT cells (mostly γδT1 cells) was undiminished with the exception of splenic Vγ1^+^ γδT cells (**Figure 3D** and **Figure S4**). Therefore, these results indicate that Syk is specifically required for the development of γδT17 cells but is largely dispensable for other subsets of γδT cells, including γδT1.

### Amelioration of skin inflammation in Syk-cKO mice

**Figure 4:**
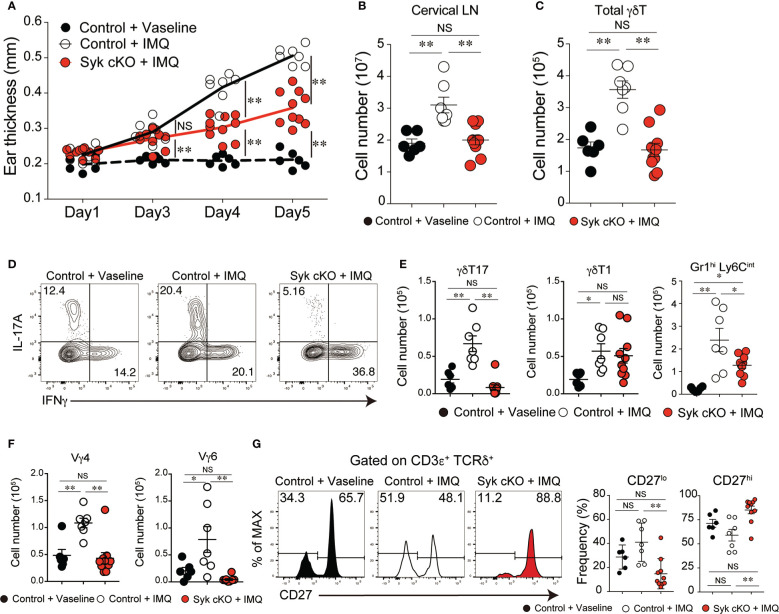
Amelioration of imiquimod (IMQ)-inducible dermatitis in Syk-cKO mice. Control (Cd127-cre Syk flox/wt) and Syk-cKO (Cd127-cre Syk flox/flox) mice were treated daily for 5 days with IMQ or Vaseline applied onto the ear. On day 5, the cervical lymph node (cLN) was isolated and analyzed. **(A–C)** The kinetics of ear swelling **(A)**, the number of total cLN cells **(B)** and the number of γδT cells in the cLN **(C)** are shown. **(D)** Intracellular staining of IL-17A and IFNγ for γδ T cells in the cLN from mice shown in **(A–C)**. **(E)** The number of γδT17, γδ T1 and Gr1^+^ CD11b^+^ cells in the cLN. **(F)** The number of Vγ4^+^ and Vγ6^+^ γδ T cells in the cLN. **(G)** Flow cytometric profiles of CD27 expression in total γδT cells from indicated mice. Graphs show the frequency of CD27^lo^ or CD27^hi^ γδT cells. Data were obtained from 6 to 10 mice in each group from two independent experiments. All data represent the mean ± SEM. *P < 0.05 and **P < 0.01, by 1-way ANOVA. NS, not significant

The accumulated evidence demonstrates that γδT17 cells play a central role in the pathogenesis of psoriatic dermatitis (1). We examined the significance of the role of Syk in the inflammatory condition by use of an imiquimod (IMQ)-induced psoriasis model. IMQ-treated control mice exhibited an increase in ear skin thickness, along with a robust expansion of cervical lymph node (LN) cells, including γδT cells (**Figures 4A**– **C**). However, Syk-cKO mice treated with IMQ exhibited a significant reduction in skin inflammation and an unaltered number of cervical LN γδT cells (**Figures 4A**–**C**). Furthermore, the IMQ-induced expansion of γδT17 cells in the cervical LN was not observed in Syk-cKO mice, while the γδT1 cell as well as αβT cell number was unchanged between the control and Syk-cKO mice (**Figures 4D, E** and **Figure S5**). The IL-17 produced in the skin promotes the recruitment of neutrophils to draining LN and dermis (32, 33). At the steady state, there are no obvious changes in the number of Gr-1^hi^ Ly6C^int^ cells that contain neutrophils (34) in the spleen and lung from Syk-cKO mice (**Figure S1B**). However, the number of Gr-1^+^ Ly6C^int^ cells in cervical LN was significantly elevated in the IMQ-treated control mice, but not in Syk-cKO mice (**Figure 4E**). Consistent with this, the increase of Vγ4^+^ and Vγ6^+^ γδT cells upon IMQ treatment was also abrogated in Syk-cKO mice (**Figure 4F**). In addition to this, the proportion of CD27^lo^ γδT cells characteristics of γδT17 cells (35) was significantly reduced in Syk-cKO mice (**Figure 4G**). These results indicate the requirement of Syk for the γδT17-mediated inflammatory responses.

## Discussion

In this study, we generated lymphoid lineage-specific Syk-deficient mice to study the role of Syk in the thymic development of γδT cells and γδTCR signaling. A large proportion of the γδT cells in the Syk-cKO mice was found to be in the early differentiation stage prior to γδT-lineage commitment, as indicated by the substantial expression of Rag genes and pTα. Syk-cKO mice also displayed a complete loss of γδT17 cell subsets and an altered γδTCR repertoire. These alternations in Syk-cKO mice resulted in a significant amelioration of skin inflammation. These results indicate that the Syk-mediated γδTCR signaling drives both γδT cell commitment and the γδT17 differentiation program.

Previous studies on the function of Syk in T cell development encountered difficulties because systemic Syk-deficient mice are perinatally lethal (19, 20) and T cell development requires *in vivo* thymus activity (8). We have previously investigated γδT cell development in fetal and neonatal Syk-deficient mice, but the requirement of Syk for γδT cell development in the adult stage was inadequately addressed (18, 36). Some pioneering studies using embryonic chimera mice or hematopoietic stem cell transplantation into immunodeficient mice demonstrated the role of Syk in epidermal and intestinal γδT cells (37, 38). However, these chimeric mouse models might not fully recapitulate the physiological conditions required for proper γδT cell development, as later studies suggested that certain γδT cell subsets require certain developmental stage-specific interplay between the progenitors and the thymic microenvironment for their full functional maturation (31, 39). The present results using Syk-cKO mice clearly demonstrate that Syk plays a pivotal role in γδT-cell lineage commitment as well as γδT17 cell priming. In the Syk-cKO mice, Vγ5Vδ1^+^ and Vγ7^+^ γδT cells normally populate in the skin and small intestine, respectively, indicating that Syk is dispensable for the development of Vγ5Vδ1^+^ and Vγ7^+^ γδT cells, most likely due to the compensatory function of Zap70 (16, 17, 40). This is consistent with previous results using fetal thymocytes from systemic Syk-deficient mice (18). Thus, the results presented here offer a conclusive answer to the long-standing question on the physiological role of Syk in γδT cell development.

It has been previously reported that γδTCR as well as preTCR induces downstream signaling in a ligand-independent manner (9, 41, 42). In Syk-cKO mice, the development of a large proportion of γδT cells were arrested at the stage of CD5^lo^ CD24^hi^ γδ precursors that still retain αβT-lineage potential. Our present findings, along with previous results, account for the molecular events in the initial phase of γδT cell development as follows: when both TCRγ and TCRδ chains are successfully rearranged and expressed in DN cells (these cells are defined as γδ-precursors), the fledgling γδTCR-CD3 complex induces Syk-mediated intracellular signaling in a ligand-independent manner. This initial TCR signaling upregulates γδT cell signature genes and downregulates those genes that are associated with TCR rearrangement and αβT cell differentiation. Thus, this sequential but rapidly unfolding process, referred to as “γδ-selection”, represents a checkpoint to ensure the generation of productive γδTCR distinct from the αβT cell lineage.

The Syk-mediated initial γδTCR signaling also involves the priming of γδT17 cell differentiation. This process is dependent on the unique ability of Syk to activate the PI3K-Akt pathway (18). It has been shown that γδT17 cells originate from distinct progenitors identified as cKit^-^CD24^hi^CD25^-^CD44^+^Sox13^+^ and this progenitor population is generated independently of γδTCR signaling (43). Thus, Syk-mediated γδTCR signaling may play a role in driving the progenitor cells to complete the differentiation program towards γδT17 cells. A recent study demonstrated that mice bearing mutations in CD3ε with attenuated γδTCR signaling exhibited decreased generation of γδT17 cells in the thymus (44). The weakened γδTCR signaling in this mouse strain is due to the lack of recruitment of adaptor protein Nck to the γδTCR-CD3 complex, indicating the requirement of Nck-mediated γδTCR signaling for γδT17 cell differentiation. Future studies are needed to determine how Nck and Syk cooperate for the activation of the PI3K-Akt pathway in order to drive γδT17 cell differentiation program.

A previous study has demonstrated that not only γδT cells but also RORγt^+^ ILCs contribute to the initiation of IMQ-induced psoriasis-like dermatitis (45). ILCs express NKp44 and NKp46 that is capable of coupling with immunoreceptor tyrosine-based activation motifs (ITAM)-containing molecules such as CD3ζ, DAP12, FcRγ. Either Syk or Zap70 is recruited to these ITAM-containing molecules (46), and may mediate the activation of intracellular signals in ILCs. In Syk-cKO mice, ILCs are expected to lack Syk alleles since ILCs are cells downstream of common lymphoid progenitors (CLP). Although quantitative and/or qualitative changes in ILCs from Syk-deficient mice have not been explored, it is possible that Syk is required for the exertion of ILC function. Because of this, we could not completely exclude the possibility that the amelioration of IMQ-induced dermatitis observed in Syk-cKO mice is caused by not only defects of γδT17 cells but also impairments of ILCs. In addition, our Syk-cKO mice have impairment of B cell development, which may also influence the induction of inflammatory responses. The use of *Tcrd*-driven Cre-expressing mice (47) would overcome this experimental limitation and allow a more rigorous elucidation of the role of Syk in early γδT cell development.

We detected a modest amount of γδT17 cells from the IMQ-treated Syk-cKO mice despite an almost complete lack of γδT17 cells in Syk-cKO mice at the steady state. Papotto et al., have previously shown that γδT17 cells can be induced from naïve γδT cells and/or expanded in LNs under the experimental autoimmune encephalomyelitis (EAE)-condition (48). Thus, it is likely that the γδT17 cells that emerged under the IMQ-condition are peripherally induced and/or expanded cells. Considering the fact that Zap70 is expressed on mature γδT cells and TCR signal enhances γδT17 differentiation *in vitro* (48), it is strongly suggested that Zap70, but not Syk, plays a central role in the regulation of γδTCR signal transduction for the peripheral induction and/or expansion of γδT17 cells.

In conclusion, it is shown that Syk plays a central role in both γδT cell commitment and γδT17 cell differentiation. These findings provide better insight into the molecular mechanisms underlying the effector determination of γδT cells and suggest that the Syk-mediated signaling pathways might be a therapeutic target in γδT17-dependent inflammatory diseases.

## Data availability statement

The original contributions presented in the study are included in the article/**Supplementary Material**. Further inquiries can be directed to the corresponding author.

## Ethics statement

The animal study was reviewed and approved by Institutional Animal Care and Use Committee of the University of Tokyo.

## Author contributions

RM and TNi designed and performed most of the experiments. TNa performed the experiments. RM, TNi and HT interpreted the results and wrote the manuscript. HT supervised the project. All authors contributed to the article and approved the submitted version.

## Supplementary material

Supplementary Figure 1 Peripheral B cells, NK cells and neutrophiles in Syk-cKO mice. **(A)** Representative flow cytometric profiles of B220 and NK1.1 expression in spleen and lung cells. The graphs depict the number of B cells (B220^+^) and NK cells (NK1.1^+^). **(B)** Representative flow cytometric profiles of Gr-1 and Ly6C expression in spleen and lung cells. The neutrophils were distinguished based on the cell surface expression of Gr-1 and Ly6C according to a previous report by Cunin et al. (34). The graphs depict the number of neutrophils (Gr-1^+^ Ly6C^int^). In **(A, B)**, 5 to 8-week-old control mice (Cd127-cre Syk flox/wt, n = 4) or Syk-cKO mice (Cd127-cre Syk flox/flox, n = 6) were used. All data represent the mean ± SEM of four independent experiments. *P < 0.05 and **P < 0.01, by unpaired t-test.

Supplementary Figure 2 Frequency and number of DN cell subpopulation in Syk-cKO mice. **(A, B)** The number **(A)** and frequency **(B)** of CD44^+^ CD25^–^ (DN1), CD44^+^ CD25^+^ (DN2), CD44^–^ CD25^+^ (DN3) and CD44^–^ CD25^–^ (DN4) cells in CD4^–^ CD8^–^ thymocytes from 5 to 8-week-old control mice (Cd127-cre Syk flox/wt, n = 6) or Syk-cKO mice (Cd127-cre Syk flox/flox, n = 7) are shown. All data represent the mean ± SEM of four independent experiments. All data represent the mean ± SEM of four independent experiments. *P < 0.05 and **P < 0.01, by 1-way ANOVA.

Supplementary Figure 3 Cell surface expression of CD3ε and TCRδ in Syk-deficient thymic γδT cells. The mean fluorescence intensity (MFI) of CD3ε and TCRδ as gated in **Figure 1E** is shown. 4 to 6-week-old control mice (Cd127-cre Syk flox/wt, n = 6) or Syk-cKO mice (Cd127-cre Syk flox/flox, n = 7) were used. All data represent the mean ± SEM of three independent experiments. *P < 0.05 and **P < 0.01, by unpaired t-test.

Supplementary Figure 4 The frequency of γδT cell subsets in the thymus and peripheral tissues. Graphs show the frequency of Vγ1^+^, Vγ4^+^, Vγ5Vδ1^+^, Vγ6^+^ and Vγ7^+^ γδT cells in the indicated tissues (n = 4 to 6). All data represent the mean ± SEM of three independent experiments. *P < 0.05 and **P < 0.01, by unpaired t-test.

Supplementary Figure 5 αβT cells and B cells in IMQ-treated Syk-cKO mice. Representative flow cytometric profiles of B220 and TCRβ expression in cervical lymph node (cLN) from IMQ- or Vaseline-treated mice as shown in . The graphs depict the number of CD4^+^ T cells (CD4^+^ TCRβ^+^), CD8^+^ T cells (CD4^+^ TCRβ^+^) and B cells (B220^+^). Vaseline-treated control mice (Cd127-cre Syk flox/wt, n= 6), IMQ-treated control (n = 7) and IMQ-treated Syk-cKO (Cd127-cre Syk flox/flox n = 10) was used. All data represent the mean ± SEM of two independent experiments. *P < 0.05 and **P < 0.01, by 1-way ANOVA.

Supplementary Figure 6 List of primers for quantitative PCR used in this study.
