# Supplementary figures and images for: Spleen tyrosine kinase mediates the γδTCR signaling required for γδT cell commitment and γδT17 differentiation
Source: Front Immunol. 2023 Jan 12;13:1045881. doi: 10.3389/fimmu.2022.1045881 (PMC9878111; doi:10.3389/fimmu.2022.1045881)

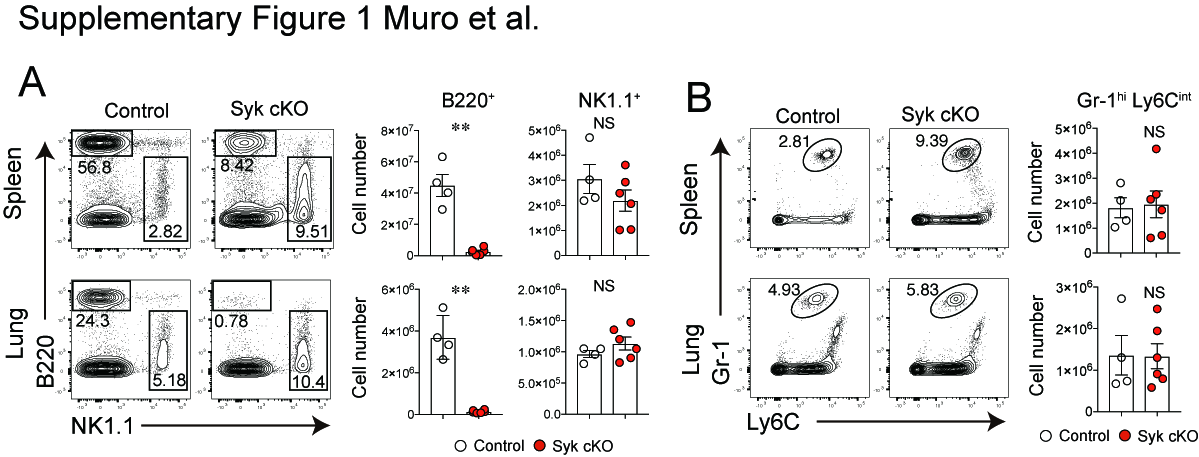

Supplement: Supplementary Figure 1 — Peripheral B cells, NK cells and neutrophiles in Syk-cKO mice. (A) Representative flow cytometric profiles of B220 and NK1.1 expression in spleen and lung cells. The graphs depict the number of B cells (B220+) and NK cells (NK1.1+). (B) Representative flow cytometric profiles of Gr-1 and Ly6C expression in spleen and lung cells. The neutrophils were distinguished based on the cell surface expression of Gr-1 and Ly6C according to a previous report by Cunin et al. (34). The graphs depict the number of neutrophils (Gr-1+ Ly6Cint). In (A, B), 5 to 8-week-old control mice (Cd127-cre Syk flox/wt, n = 4) or Syk-cKO mice (Cd127-cre Syk flox/flox, n = 6) were used. All data represent the mean ± SEM of four independent experiments. *P < 0.05 and **P < 0.01, by unpaired t-test. [file Image_1.tif]

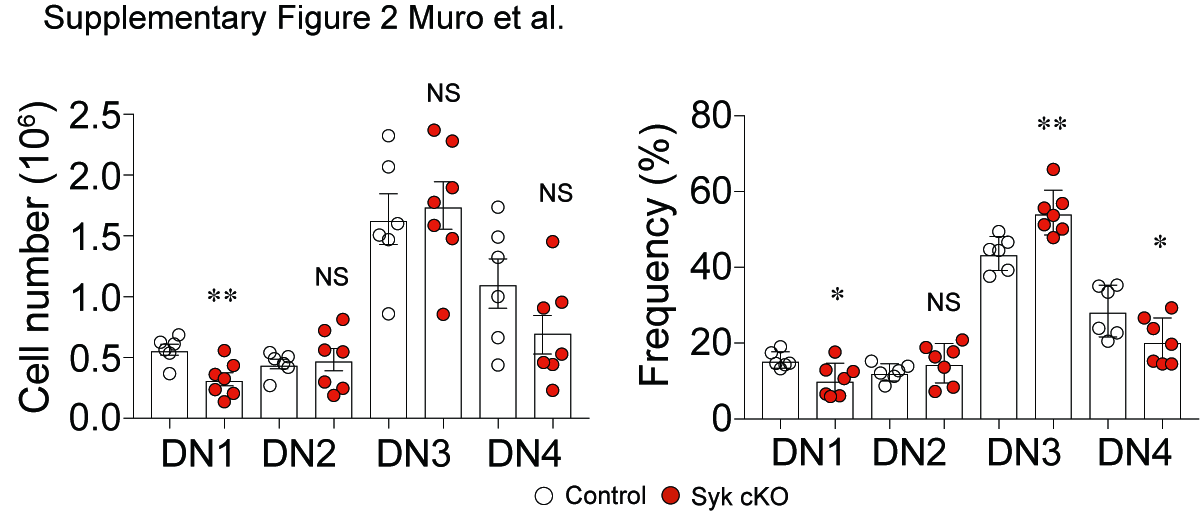

Supplement: Supplementary Figure 2 — Frequency and number of DN cell subpopulation in Syk-cKO mice. (A, B) The number (A) and frequency (B) of CD44+ CD25– (DN1), CD44+ CD25+ (DN2), CD44– CD25+ (DN3) and CD44– CD25– (DN4) cells in CD4– CD8– thymocytes from 5 to 8-week-old control mice (Cd127-cre Syk flox/wt, n = 6) or Syk-cKO mice (Cd127-cre Syk flox/flox, n = 7) are shown. All data represent the mean ± SEM of four independent experiments. All data represent the mean ± SEM of four independent experiments. *P < 0.05 and **P < 0.01, by 1-way ANOVA. [file Image_2.tif]

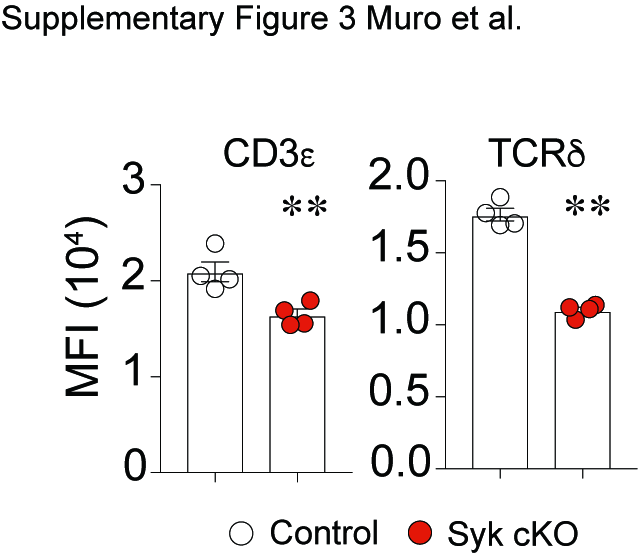

Supplement: Supplementary Figure 3 — Cell surface expression of CD3ε and TCRδ in Syk-deficient thymic γδT cells. The mean fluorescence intensity (MFI) of CD3ε and TCRδ as gated in Figure 1E is shown. 4 to 6-week-old control mice (Cd127-cre Syk flox/wt, n = 6) or Syk-cKO mice (Cd127-cre Syk flox/flox, n = 7) were used. All data represent the mean ± SEM of three independent experiments. *P < 0.05 and **P < 0.01, by unpaired t-test. [file Image_3.tif]

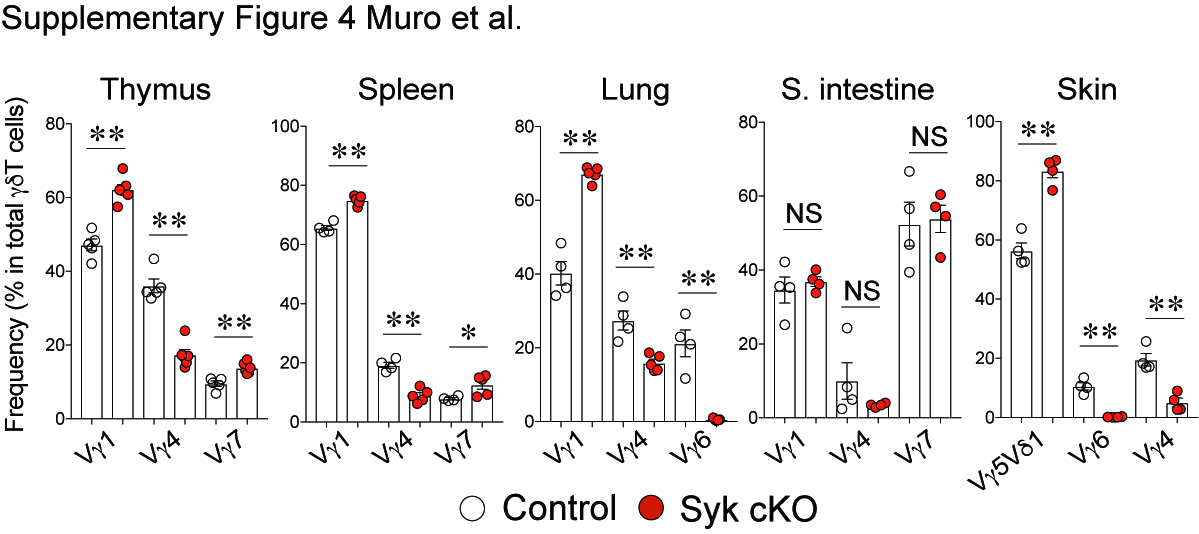

Supplement: Supplementary Figure 4 — The frequency of γδT cell subsets in the thymus and peripheral tissues. Graphs show the frequency of Vγ1+, Vγ4+, Vγ5Vδ1+, Vγ6+ and Vγ7+ γδT cells in the indicated tissues (n = 4 to 6). All data represent the mean ± SEM of three independent experiments. *P < 0.05 and **P < 0.01, by unpaired t-test. [file Image_4.tif]

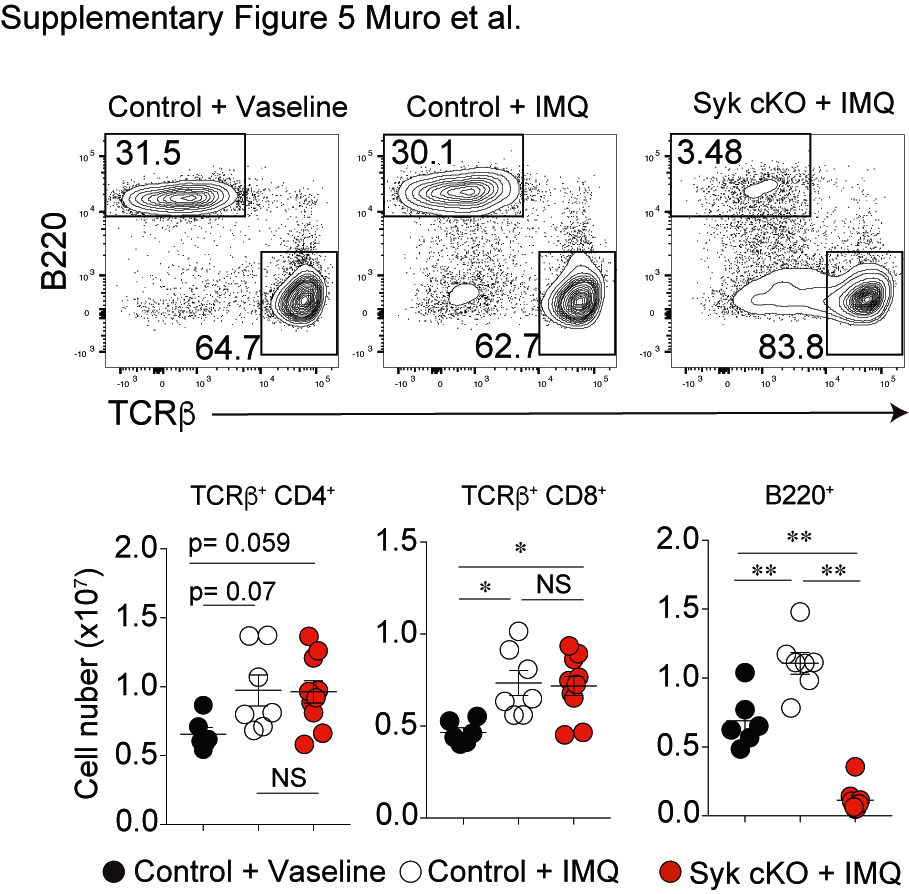

Supplement: Supplementary Figure 5 — αβT cells and B cells in IMQ-treated Syk-cKO mice. Representative flow cytometric profiles of B220 and TCRβ expression in cervical lymph node (cLN) from IMQ- or Vaseline-treated mice as shown in . The graphs depict the number of CD4+ T cells (CD4+ TCRβ+), CD8+ T cells (CD4+ TCRβ+) and B cells (B220+). Vaseline-treated control mice (Cd127-cre Syk flox/wt, n= 6), IMQ-treated control (n = 7) and IMQ-treated Syk-cKO (Cd127-cre Syk flox/flox n = 10) was used. All data represent the mean ± SEM of two independent experiments. *P < 0.05 and **P < 0.01, by 1-way ANOVA. [file Image_5.tif]

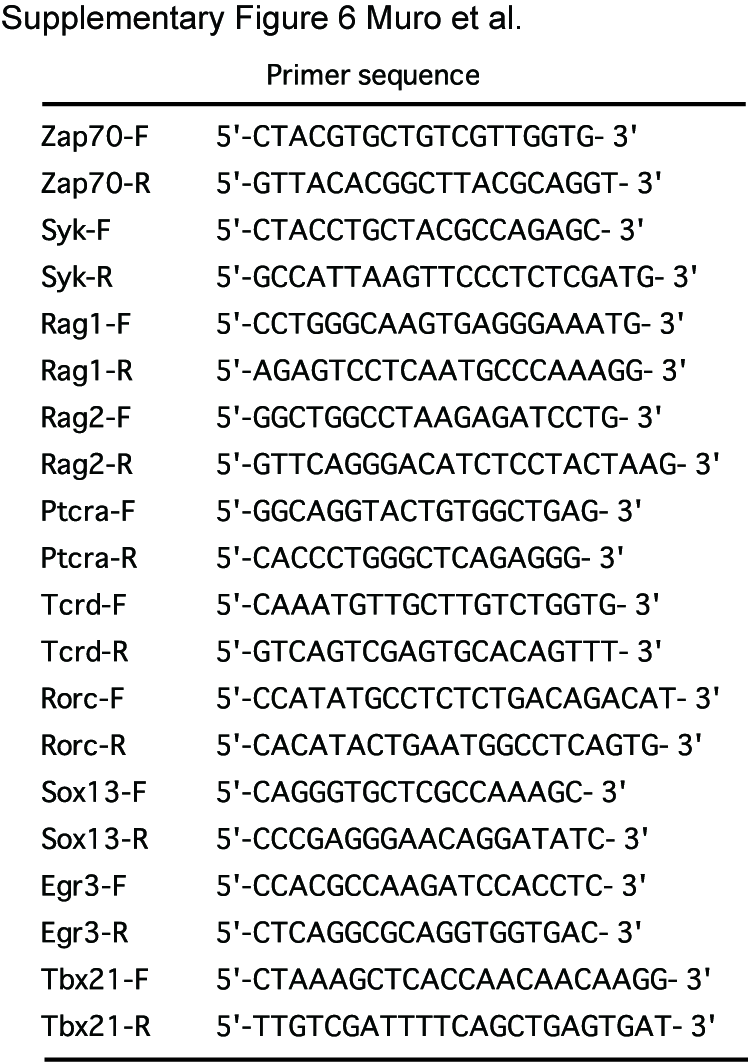

Supplement: Supplementary Figure 6 — List of primers for quantitative PCR used in this study. [file Image_6.tif]
